# International medical students’ expectations and worries at the beginning of their medical education: a qualitative focus group study

**DOI:** 10.1186/s12909-016-0549-9

**Published:** 2016-01-28

**Authors:** Daniel Huhn, Julia Huber, Franziska M. Ippen, Wolfgang Eckart, Florian Junne, Stephan Zipfel, Wolfgang Herzog, Christoph Nikendei

**Affiliations:** Centre for Psychosocial Medicine, Department of General Internal Medicine and Psychosomatics, University Hospital Heidelberg, Thibautstraße 2, 69115 Heidelberg, Germany; Institute for History and Ethics in Medicine, Ruprecht-Karls-University Heidelberg, Heidelberg, Germany; Department of Psychosomatic Medicine and Psychotherapy, University Hospital Tubingen, Tubingen, Germany

**Keywords:** International medical students, Expectations and fears, Focus group study

## Abstract

**Background:**

The number of international students has increased substantially within the last decade. Due to cultural barriers, this specific group faces diverse challenges. In comparison to German colleagues, international medical students perform significantly lower in clinical examinations and exceed the average duration of study; they suffer from personal distress as well as insufficient support. Within the present study, their individual perspectives, expectations, hopes and fears were examined.

**Methods:**

Four focus groups with first-year international medical students (*N* = 16) were conducted in October 2013. Each 60- to 90-min discussion was audiotaped, transcribed and analysed using qualitative methods.

**Results:**

International medical students go abroad in search of good study-conditions. For the choice of place of study, affordability, social ties as well as an educational system following the achievement principle are decisive factors. While contact with German-students and other international students is seen as beneficial, international medical students are most concerned to encounter problems and social exclusion due to language deficits and intercultural differences.

**Conclusions:**

Facilitating the access to university places, the provision of financial aid and, moreover, social support, nurturing cultural integration, would greatly benefit international medical students. Hereby, the establishment of specific medical language courses as well as programs fostering intercultural-relations could prove to be valuable.

## Background

Globalization, one of the big issues at present, does not stop at student education. Over the last decade the number of students studying in foreign countries has increased by 77 per cent, with a peak of almost 3.7 million people in 2009 [1]. Thus, international students have become a highly coveted and increasingly appreciated immigrant group [2]. Several facts about this specific group have been compiled so far: Australia, the United Kingdom, Austria, Switzerland and New Zealand have the highest percentages of international students among their tertiary enrolments. In absolute terms, the US, the UK, Australia, Germany and France account for nearly half of them, while the largest numbers of international students come from China, India and South Korea. Asian students represent more than 50 % of foreign students studying abroad, with European countries at the forefront of preferred study locations (38 %) followed by North America (23 %) [1]. In a comparison of OECD-countries, health and welfare studies are the fourth most attractive field among international students [1]. Accounting for up to 15 % of all medical students and with more than 2.000 young people starting studies each year, medicine and health care sciences are the seventh most popular subjects among international students in Germany [3].

Extant literature has shown that international students face diverse challenges as a result of language and cultural barriers [4, 5]. They are often confronted with a lack of social support [6], alienation and homesickness [7, 8], face financial as well as academic difficulties [7] and sometimes even have to cope with racial discrimination [8]. It could be documented for the specific field of medical education that international medical students suffer from higher levels of stress [9], reduced quality of life [10] as well as the loss of social contacts [11]. Besides, international medical students achieve lower results in pre-clinical written tests [12], in clinical examinations [13–15] as well as state examinations [12], and show an extended duration of study [16] as well as higher drop-out rates [11, 17].

However, apart from these quantitative results, only little is known about international students’ individual perspectives and feelings during their transition to studying overseas. So far, the motives behind leaving their home countries to study abroad as well as their reception at respective universities remain largely unclear. Little is known about their expectations regarding their forthcoming studies, their prospective lives in a foreign country or their hopes and anxieties for the future. A better understanding of this target group could be beneficial for the improvement of integration and support efforts as well as for the identification of further research areas. Therefore, the aim of the current study was to determine international students’ expectations and familiarisation processes at the beginning of their studies and during the transition from life in their home country to the realities of living as international students. To this end, international medical students were asked to participate in focus group interviews on a voluntary basis at the beginning of their studies. Here, they were granted the opportunity of reflecting their expectations, hopes and fears concerning their future studies. Results were analysed to assess international students’ individual perspectives and to identify crucial aspects for which an improvement of conditions would be promising.

## Methods

### Participants

Each year approximately 360 students start studying human medicine in Heidelberg, with up to 10 % being international students coming to Germany for the first time for their studies. Having been born and raised abroad, they have received secondary education outside of Germany. However, the university’ language of instruction is German in all courses. Please see Nikendei et al., 2009 [18] for a comprehensive overview of the German medical curriculum. Therefore, international students must prove very good German language skills (at least B2-level language skills) in their application. For these international students, a specific peer-led tutorial – the Heidelberg Tutorial for international Medical Students (HeiTiMed) [19] – is offered supporting students in their pre-clinical examinations in the first two semesters by coaching them for examinations ahead. Students were invited to participate in the study within the framework of this tutorial at the beginning of the first semester. Participation in the study was voluntary and there were no entrance requirements.

Sixteen international first-year medical students (9 female, 7 male, M _Age_ = 19.9 years, SD _Age_ = 1.8 years) participated in the study. Further details of the participants are summarized in Table 1.

**Table 1:** Characteristics of the international students participating in the focus group interviews (*n* = 16)

Number of participants		16
Sex	Female	9
	Male	7
Mean age [years]		19.9 ± 1.8
Origin	Europe	5
	Middle East	4
	East Asia	4
	Southeast Asia	2
	Latin America	1

### Focus group interviews

Consenting international students were allocated to focus groups consisting of three to five participants. A new focus group was opened until the content saturation point was reached [20]. As no significant new ideas emerged during the fourth focus group, no further sessions were organized [21]. All focus groups took place shortly after participants had started their medical studies.

The aim of the focus group interviews was to explore the subjective perception, judgment and expectations of international medical students at the beginning of their studies. In a qualitative approach, focus groups are appropriate for exploring participants’ views as well as the underlying notions and considerations [21]. Group discussions are especially suitable for research areas focusing on participants’ diverse views as well as emotional experiences [22].

To counteract potential biases due to social pressure inhibiting students to express their opinions openly, the moderator emphasized the value of differing opinions and views and asked the participants to note their thoughts on a topic before group discussion started. Four one and a half-hour interviews were held on different days, moderated by one of the authors (DH), who is an experienced moderator of small groups. To guarantee consistency between the focus groups, a question route was implemented [21], for more details see Table 2. Interviews were recorded and subsequently transcribed.

**Table 2:** Question route used by the moderator in guiding focus group discussions

1. What were your motives to start studying medicine abroad?	
2. Have you already built up a social network at your new home?	
3. Which expectations or worries do you have concerning your future studies with respect to the existing cultural differences?	
4. In your opinion, what is important for the successful integration of international students?	
5. In your opinion, is there any need for improvement of the existing conditions?	

### Analysis of the transcripts

The verbatim-transcribed discussions were analysed via qualitative content analysis using the software MaxQDA (version 11, VERBI GmbH, Berlin). In accordance with guidelines for qualitative inductive content analysis [23], an open coding of all discussions was conducted first to search for recurring topics. Single or a few sentences were identified as a code, representing the most elemental unit of meaning [24]. Next, the codes were summarised into relevant themes for each participant. The assignment of respective codes to specific themes was conducted by three independent analysts (DH, JH and FJ) and subsequently discussed to reach consensus and, if required, adjusted. In a final step, themes were grouped into five relevant categories.

As all interviews were conducted in German language, obtained data were also analysed in German and subsequently translated into English language.

### Ethics

Study participation for international students was voluntary. All students were adequately informed about the purpose of the study and granted anonymity and confidentiality regarding their data. In addition, written consent was obtained from all participants. Ethics approval was granted by the ethic committee of the University of Heidelberg. The study was conducted according to the Declaration of Helsinki (64th WMA General Assembly, Fortaleza, Brazil, October 2013).

## Results

### Main categories and themes resulting from qualitative analysis

With regard to the qualitative analysis of the transcripts, 245 relevant single statements of interviewees were identified within the structured focus group discussions. From these, 21 themes and five main categories were derived. The main categories included (A) motivation for studying medicine abroad, (B) established social network at the place of study, (C) expectations/worries concerning the near future, (D) determinants for successful integration, and (E) need for action to improve conditions for international students. Each of these main categories (A to E) contained 2–6 themes (i.e. A.1 to A.6). However, the main categories should not be understood as completely independent from each other as some cover similar content.

### Definition of themes

In the following, we provide definitions for the themes belonging to the main categories.

### Category A: Motivations for studying medicine abroad (57 quotations)

#### Theme A.1: Quality of the study program (14 quotations)

For international students an excellent level of teaching as well as the universities’ international reputation were relevant criteria for their choice of study place as expressed by one international student, who was convinced that “Medical study programs in Germany seem to be very good, […] ranking in the first places most of the times”.

#### Theme A.2: Prior reference to the country chosen (14 quotations)

International students indicated that they had different points of contact with the country chosen before going abroad. For example, one student indicated, that his “brother had also previously studied medicine in Germany” so that he “already had some sort of connection”. Others had already “participated in exchange programs with German schools” in earlier years, which gave them the “opportunity to get to know to German culture and the language more closely” also reflecting a principal interest in the destination’s language and culture.

#### Theme A.3: Study conditions (13 quotations)

“The cost-benefit ratio in Germany is very good. Yes, for example, there are better courses for Medicine in America or Japan, but they cost a whole lot” one student stated regarding the fact that studying in Germany – in contrast to most other countries – is virtually free of charge, hence leading to highly eased framework conditions. Besides, the application process in the country chosen was perceived to be relatively easy as international students are “marked up equally with the German applicants”. They applied “just like the Germans” as “qualification grades were converted so [that] it was a fairly simple process.” Hence, students usually encounter only minor hurdles in the application process and the study conditions in Germany are seen to be better than elsewhere.

#### Theme A.4: Studying abroad (7 quotations)

The international students’ motivation for studying abroad was both to experience adventure and simultaneously promote their autonomy and personal development. This “special experience, an adventure” was expected to make international students “more mature and independent from [their] home country” and was seen as a chance to “perhaps also test [the] own limits a little bit”.

#### Theme A.5: Achievement principle vs. corruption (5 quotations)

The quote: “What you know has its value, here. And not from which family you come or how much money you have” reflects the importance and the value of the principle of meritocracy in the country chosen. “Special contacts are irrelevant here, since there is almost no corruption” another student added. Students seem to be attracted by the fact that they can rely on their own resources and are not dependent on others with their own effort translating to personal success.

#### Theme A.6: Spontaneity / Random (4 quotations)

Some of the students stated that there were no specific reasons for their choice of study place. Their choice was more likely to be attributable to chance as mentioned by one of the international students: “Actually, it was a coincidence that I came to Germany”.

### Category B: Established social network at the place of study (57 quotations)

#### Theme B.1: “Study-related network” (21 quotations)

International students had already made first social contacts. Due to the time they had spent at university and in the clinic, most of these contacts were with student peers. Students felt to have “so little time since [they] had to study all day long” asking themselves “who else [they] should […] meet with than [other] colleagues?” with their main focal point being the university.

#### Theme B.2: “Private network” (8 quotations)

Some of the international students pointed out that they enjoyed having “friends not only at the university but also in other contexts”. Some reported having friends among “neighbours, roommates […] and people from sports clubs” as well as still keeping in contact to “a German guest-family with whom [they] have lived for more than two months”.

#### Theme B.3: “International acquaintances” (16 quotations)

The most visible social network was seen in other international students with whom they spent a lot of their time. The fact that international students “have more foreign friends” might root in the circumstance that they “have the same problems and face the same difficulties,” and therefore share important commonalities in their everyday lives. Despite being from completely different cultures, they still shared the similarity of being strangers in Germany creating a lot of common ground. Furthermore, due to the university’s specific offers for international students, they had already palled up “with other international students in preliminary courses before starting studies”.

#### Theme B.4: “Local acquaintances” (12 quotations)

Contact to local people was indicated as “pretty important […] because someday we need to talk to a German in German”. Acquaintances with Germans were experienced as helpful for practicing the German language as well as for familiarizing with German culture.

### Category C – Expectations / Worries concerning future times (64 quotes)

#### Theme C.1: “Difficulties with studies because of language deficits” (29 quotations)

Not all of the international students felt able to cope with upcoming examinations due to existing language deficits. They reported to be “scared […], [and to] have feelings of failure and uncertainty regarding the exams” as they still had problems understanding the new language, especially in the first months. Comparing themselves to their German peers, they expected to need more time processing language related issues and also anticipated having to work harder and having to invest more time. As one student stated “where German students probably need 10 min to read three pages in a book, I probably need 3 h”. Another obstacle could appear if “patients talk in a dialect or says phrases [they have] never heard of in language school”. Due to the language barrier, international students were concerned about failing in such situations, especially when confronted with colloquial language or dialects. Some international students were even concerned to experience problems with integration in the student group as they had “a hard time understanding German students”, for example, when they “talk too fast […] and sometimes very colloquially”.

#### Theme C.2: “Experiencing intercultural differences” (13 quotations)

Another apprehension that emerged among international students was that they may face problems due to intercultural differences. Regarding the concerns of failing in certain social situations due to their unfamiliarity with cultural scripts for the respective situation, students thought it would be helpful “to ask Germans about specific cultural facts” since they did “not always understand everything”. Furthermore, they expressed concern to be confronted with prejudices and stereotypes simply because of being foreign. One student stated to be “a little bit afraid that people will meet me with prejudice here” because he came “from Turkey and Turks are not the most popular population in Germany in general”.

#### Theme C.3: “Problems in social situations” (12 quotations)

Concerning social situations, some international students stated that they might have problems with Germans due to the Germans’ more reserved nature or because of a feeling of exclusion. The concern was that “if you just wait and don’t try to start a relationship or improve it, then you will just get nowhere with your social life” and hence they themselves “always [had] to make the first move”. However, other students held an opposite opinion, expecting German people to be open and welcoming since they had already made the experience that “people smile and that they are friendly”.

#### Theme C.4: “Hope for integration in a largely fair system” (6 quotations)

In light of the weak corruption rate in Germany, international students expected to encounter a largely "fair" system, in which similar conditions were available to all regardless of wealth and status. Students felt that in other countries better opportunities were often given in correlation with “the more money you have” which is seen as “very unfair for the people who do not have much money.”

#### Theme C.5: “Confusion regarding the German health care system” (4 quotations)

International students felt that “everyone expects [them to] already know everything about the health care system” and expressed concern that this lack of knowledge about “how it all works” could lead to irritations since students felt that they were expected to be more professional.

### Category D: “Factors for successful integration” (32 quotations)

#### Theme D.1: “External factors” (17 quotations)

International students saw a number of ways to facilitate the universities’ integration of minorities. Fortunately, some supportive offers already exist, like specific exam preparation courses; still, the universities could try harder to create conditions raising the awareness for intercultural issues, as for example, “student parties on various topics that are somewhat culturally themed”. Similar offers would represent an excellent opportunity to “somehow showcase your culture in front of local students”. Other ideas seen to promote integration were related to initiatives at political level (e.g. granting visa etc.). However, conditions in this field were already seen to be fairly good as one student stated “and because I study medicine, I got a permanent visa which I thought was really good”. Local people’s behaviour was also mentioned by a few international students. “Successful integration” was considered to take place “when foreigners are perceived as comrades, or as other students and not as a foreigner”. Hence, local people’s open-mindedness could play an important role in how successful the integration is experienced.

#### Theme D.2: “Internal factors” (15 quotations)

In addition to the external factors listed before, international students also discussed personal attitudes such as curiosity, openness or shyness as indicators of successful integration. Regarding this, it would be “best to confront [yourself] with other people” to draw comparison and, “if you have issues not to bottle them up”.

### Category E: “Need for action to improve conditions for international students” (23 quotations)

#### Theme E.1: “Need for improvement through university” (18 quotations)

Asked about the need for improvement, many international students stated that universities could simplify the process of application, could help international students with exam preparations or could create conditions to raise the awareness for intercultural issues. Regarding the latter, “organized social activities on cultural issues” are seen as a good possibility for international students to “explain how it works in [their] home country”; perhaps local students “would also be curious about this”.

#### Theme E.2: “Need for improvement in contact with Germans” (5 quotations)

Some international students indicated that an increased understanding for the difficulties they faced would be helpful feeling that “empathy is missing a little” in this regard with as local students having problems understanding that “it’s a bit difficult for the foreign students”.

## Discussion

Aim of the current study was to elucidate the feelings and expectations as well as the hopes and fears of international medical students at the beginning of their studies in regard to their current situation and future perspective while studying abroad.

Considering the motives prompting international students to start studying medicine abroad, we revealed that the presumed quality of the study program, the renown attached to the university of interest as well as a solid position in international university rankings were decisive factors for application. Furthermore, the cost of both study and accommodation, particularly in regard to the perceived cost-benefit ratio, were further decisive elements influencing the students’ choice of place of study. High study fees are known to deter students particularly from disadvantaged socio-economic backgrounds [25]. As tuition fees in Europe are known to be moderate in comparison to the US, Canada or Australia [26], the choice of a European university comes as no surprise.

Prior references, like relatives, close friends or an already existing interest in the local culture or language also played an important role in their choice. Largely alleviating psychological adjustment, the high relevance of social ties for the successful process of acculturation of international students is well known [27]. Moreover, international students endeavoured to gain a university placement in countries with marginal levels of corruption and nepotism, as found in the country chosen, where the rules on the recognition of the results obtained and diploma awarded follow a well-established principle of achievement. Accordingly, in sharp contrast to institutions of higher education practicing professional misconduct, simultaneously providing students with less incentive to learn [28], the students’ feeling of self-efficacy may increase here as they are able to progress through personal merit rather than through personal connections. The experience of adventure and autonomy was named as a further motivational factor behind the students’ decision to study abroad, with the place of study playing an inferior role as long as it was away from home.

Considering international students’ social networks, study-related contacts as well as relations with other international students were most prominently reported to have significant importance. This might be explained by the fact that international students spend most of their time on the campus naturally forming relations with colleagues from university. Moreover, catalysed by the simple fact of having the commonality of being foreigners abroad, specific preliminary courses for international students facilitated the formation of acquaintances among each other. When confronted with the aforementioned lack of social support, alienation as well as homesickness [6–8], the international students reported to find some relief by conversing with like-minded people, possibly experiencing similar difficulties.

In relation to expectations and worries concerning the future, difficulties with their studies because of language deficits seemed to be the most prominent concern. The international students were concerned about failing examinations, encountering communication problems in particularly in contact with patients and not being able to shoulder the expected higher workload through the required higher investment of time for studies compared to German colleagues. With several studies that have shown international students’ lower examination performances compared to local students in mind [12, 15, 29], these concerns seem to be justified to some extent. However, linguistic differences are mostly discussed as reasons for this performance distinction [13]. In addition, a small number of interviewed students was concerned about not being able to socialise adequately with local -students due to the language barrier.

Furthermore, international students expressed the concern of encountering difficulties due to intercultural differences – a phenomenon often reported by minority groups [30]. On the one hand, they feared failure in social situations as they were not yet adept in local cultural scripts. On the other hand, they were concerned that local inhabitants might be prejudiced towards them because of their migration background. Most students expected locals to have a very reserved attitude towards foreigners and thus expressed a fear of being excluded or marginalised. However, some students reported positive experiences having encountered open and welcoming attitudes.

Alarmingly, the only positive feeling reported by students on expectations in regard to future studies was their hope of encountering a fair system in which similar conditions were available to all regardless of wealth and status. The fact that international students mainly focused on potentially negative developments, when asked about future expectations and concerns, could be seen as a further reference point for their feeling of insecurity.

International students named external as well as internal factors influencing successful integration. Among the most prominently reported external factors where specific supportive offers provided by the universities. In Germany, for example, extant literature has shown that supportive offers for international students, like specific exam preparation tutorials in the preclinical term, specific language courses as well as tandem programs which are provided by most medical faculties, seem to play an important role [31]. However, the study does not report supportive offers raising the awareness for intercultural issues through the discussion of cultural differences [31] and – to our best knowledge – no further examples of such offers can be found in extant literature. Furthermore, initiatives at political level, for example, the alleviation of visa conditions for international students in general, as well as the locals’ behaviour were named as important issues for successful integration. In addition, the international students reported a variety of personality traits, such as openness towards and curiosity for the situation of being abroad, which they felt were important internal factors helping them to overcome initial concerns

Concerning the need for action to improve conditions, we revealed that students felt that the most relevant action to improve support could be undertaken by the university itself. In light of needs expressed by the international students, universities could still do more to support their international body of students, like providing them with urgently needed linguistic assistance or by raising the awareness for the international students’ specific situation among the general body of students. Hence, lowering the hurdles for acculturation and creating a deeper mutual understanding. In summary, study abroad can be predominantly viewed as a means of academic and personal enhancement. International students go abroad in search of better study-conditions and personal adventure, tuition fees, social ties as well as an implemented principle of achievement at the place of study were the most decisive factors for their decision. While contact with German students and other international students is seen as beneficial, international medical students are most concerned to encounter problems in examinations or in the contact with patients due to language deficits. In addition, they are concerned that local people could be prejudiced due to intercultural differences rendering them socially excluded.

Facilitating the access to university places, the provision of financial aid and, moreover, social support, nurturing cultural integration, would greatly benefit international medical students. Hereby, the establishment of specific medical language courses as well as programs fostering intercultural-relations could prove to be the most important means in order to help and support international medical students. Even though programs supporting international medical students [31, 32] have already been established, the current study shows that awareness for this issue must be maintained.

During the qualitative analysis, we were intrigued by how the derived main categories and themes inter-related. One might speculate that international students’ high work load combined with their anxiety and initial language deficits could result in social withdrawal. Accordingly, further incentives for integrative support within the university-community are strongly recommended. Further research should address the interlinkage of these factors.

### Limitations

The focus group interviews’ susceptibility to bias has to be named as limitation of the present study. Although adequate measures for prevention were taken, interviewees might have been influenced by the moderator. Moreover, the voluntary participation in the study possibly led to biases in our analysis. Furthermore, due to the small number of participants, it was not possible to control for differences in participants’ nationalities during analysis.

## Conclusion

Although a variety of studies have investigated international students’ situation [33, 34], the present study is, to the best of our knowledge, the first to examine international medical students’ expectations, fears and hopes concerning their future at the very beginning of their studies. The study’s results demonstrate that international students could be supported in various ways: First, facilitating the access to university places through financial aid could be beneficial, especially for students from disadvantaged backgrounds. Second, offering specific medical language courses could alleviate international students’ situation, in particularly concerns regarding failure in clinical examinations or in contact with patients. And last, offering programs fostering intercultural-relations to all students could raise local students’ awareness for their international colleagues’ difficult situation lowering the hurdles for acculturation and creating a deeper mutual understanding and making a major contribution regarding their social integration. Further research should address the effectiveness of these aspects. Moreover, future research should not only address international students’ expectations before but also actual experiences made during their studies. Here, it would also be interesting to examine whether international students differ in their perceptions depending on their gender or cultural backgrounds.
